# The Potential for Bias across GPS-Accelerometer Combined Wear Criteria among Adolescents

**DOI:** 10.3390/ijerph19105931

**Published:** 2022-05-13

**Authors:** Maura M. Kepper, Amanda E. Staiano, Stephanie T. Broyles

**Affiliations:** 1 Prevention Research Center, Washington University in St. Louis, St. Louis, MO 63130, USA; 2 Pennington Biomedical Research Center, Baton Rouge, LA 70808, USA; amanda.staiano@pbrc.edu (A.E.S.); stephanie.broyles@pbrc.edu (S.T.B.)

**Keywords:** physical activity, adolescents, accelerometry, GPS, measurement

## Abstract

Physical activity has many health benefits, yet a large portion of our population is not meeting recommendations. Using accelerometry and global positioning systems (GPS) to accurately measure where people are active and to identify barriers and facilitators of activity across various settings will inform evidence-based policies and interventions to improve activity levels. Criteria for sufficient accelerometry data (e.g., number of days, minimum hours in a day) to accurately monitor free-living physical activity in adults and children have been widely studied, implemented, and reported by researchers. However, few best practice recommendations for researchers using GPS have been established. Therefore, this paper examined the impact of three co-wear criteria of varying stringency among a sample of children aged 10 to 16 years in Baton Rouge, Louisiana. Overall and location-based physical activity was consistent across the samples even within sociodemographic subgroups. Despite the lack of significant subgroup-specific mean differences in physical activity across the three samples, associations between sociodemographics and weight status and physical activity were significantly different depending on the device time-matching “co-wear” criteria applied. These differences demonstrate the critical impact co-wear criteria may have on conclusions drawn from research examining health disparities. There is a need for additional research and understanding of ideal co-wear criteria that reduce bias and accurately estimate free-living location-based physical activity across diverse populations.

## 1. Introduction

The benefits of regular physical activity, specifically moderate-to-vigorous intensity physical activity (MVPA), are well-acknowledged for children and adolescents, and many health behaviors track into adulthood [1,2,3]. Physical inactivity was identified as one of the four leading risk factors for non-communicable diseases, which account for 88 percent of all deaths in the United States [4]. Yet, a large proportion of youth are not meeting MVPA recommendations and are instead leading sedentary lifestyles [1,5]. People are active in many settings and locations (e.g., at school, at or near home, walking around the neighborhood, etc.), and ecological models suggest that physical activity varies across these settings based on both interpersonal (e.g., family and peer support) and individual factors (e.g., age, gender) [6,7,8]. Understanding where people are active and identifying barriers and facilitators of physical activity within those settings (e.g., access to park equipment, amount of greenspace, safety) is necessary to develop and implement effective evidence-based policies and interventions to promote activity including MVPA [9,10].

The extent of evidence for environmental determinants of physical activity has rapidly grown. This is in part due to the advancement of technology; improvements in the measurement of physical activity (e.g., objectively measured via accelerometry) provide a more accurate representation of this health behavior by measuring duration and intensity (e.g., MVPA), and advances in computer software (e.g., geographic information systems; GIS) provide the tools to measure physical environmental characteristics of the land that people inhabit. Most recently, an increasing number of researchers have begun to use portable, consumer-grade geographic positioning system (GPS) devices in conjunction with accelerometers to objectively measure spatial behaviors and how people use their built environment for physical activity [10,11]. When combined, these devices allow us to understand how certain locations influence physical activity to develop multilevel interventions that generate a duration and intensity of physical activity that results in health benefits [8,10,12].

With technological and methodological advancements, there are also new practical challenges, limitations and questions of generalizability [13]. Criteria for sufficient accelerometry data (e.g., number of days, minimum hours in a day) to accurately monitor free-living physical activity in adults and children have been widely studied, implemented and reported by researchers. For example, a minimum of 3 days (including one weekend day) with at least 10 h of waking wear time is commonly used and validated to examine children’s free-living activity [14]. However, few best practice recommendations for researchers using GPS have been established. Kerr and colleagues help researchers select GPS devices and settings, perform data collection, clean and process data and integrate data into GIS; however, there are no explicit recommendations for wear time inclusion criteria for combined GPS and accelerometry data [13]. As a result, few studies using both accelerometry and GPS report their GPS inclusion criteria and, among those that do, criteria vary widely. For example, some papers are applying more stringent criteria such as the exclusion of participant data if there were fewer than five days of data with 600 min of matched GPS and accelerometer wear [15]. Another study required two valid weekdays and one valid weekend day with at least two valid hours (at least 10% of the hour had matched data) of matched data [16,17]. By contrast, some researchers are requiring only one minute of combined GPS and accelerometry data to denote a valid day of wear [18].

As we have seen in physical activity research using accelerometry, the application of varying inclusion criteria may lead to systematic bias in study results [19,20,21]. One study has examined the impact of different GPS criteria on sample characteristics and reported sociodemographic, and likely environmental, biases among the retained sampled compared to those excluded from the analysis [22]. Yet, no studies have examined whether differences in the sample caused by differing wear time co-wear criteria for GPS–accelerometry also lead to differences in estimates of physical activity and its relationships with other variables. Therefore, this paper aims to apply various inclusion criteria previously applied in other studies and examine the impact of different samples (i.e., participants meeting the different co-wear time criteria) included for analysis on estimates of levels of physical activity as well as on relationships between physical activity and commonly-studied sociodemographic and ecological characteristics.

## 2. Materials and Methods

### 2.1. Participants and Procedures

Children aged 10 to 16 years (mean: 12.6 ± 1.9 years; 53.5% girls) were recruited from the largely urban greater Baton Rouge area to participate in the Translational Investigation of Growth and Everyday Routines in Kids (TIGER Kids) Study (USDA 3092-51000-056-04A). Consent from parents/legal guardians and assent from adolescents were obtained upon each participant’s arrival at the Pennington Biomedical Research Center Translational Research Clinic for Children. Children aged 10 to 16 years and weighing < 226.8 kg (500 pounds) were eligible to participate in TIGER Kids; children were excluded if pregnant, on a restricted diet due to illness, or for having significant physical or mental disability. 

### 2.2. Data Collection

Parents completed a demographic form that included the adolescent’s date of birth, sex, and race/ethnicity. Participant standing height and weight were measured by research staff to the nearest 0.1 cm and 0.1 kg, respectively; two measurements were taken, and the average was used for analysis (a third measurement was obtained if the two measurements differed by more than 0.5 units). During measurement, participants wore a gown and no shoes, and gown weight was subtracted to calculate final weight.

Participants were asked to wear the triaxial accelerometer (Actigraph GT3X+, Actigraph, Ft. Walton Beach, FL, USA) and the QStarz BT Q1000XT GPS data logger on an elasticized belt around their hip. Devices were synchronized by the study team at the point of delivery to ensure tracking timestamps aligned. Participants were encouraged to wear both devices 24-h per day for at least 7 days, including 2 weekend days and received regular messages to remind them to charge the GPS and to wear their devices. Participants were instructed to remove the devices during water-based activity (e.g., showering, bathing, swimming) as devices are not waterproof. Participants were asked to re-wear the devices if they did not wear the accelerometer for at least 4 days with 10 h of wear time. Data were downloaded into ActiLife software and assessed for valid wear immediately upon receipt. Data were collected during school and summer terms between 2016 and 2018. Accelerometers were initialized to 15-s epochs; whereas GPS location and velocity data were captured approximately every 5 s.

### 2.3. Data Processing

#### 2.3.1. Identifying Location as within Neighborhood

Every GPS point was identified as within or outside the participant’s home neighborhood. Home addresses of all participants were geocoded and validated with GPS data. Neighborhoods were defined using 1.2 km (¾ mile) street network buffers around the home address, which is considered an acceptable walking distance [23]. Data were stored and processed using PostgresSQL 10 version, (The PostgresSQL Global Development Group, University of California at Berkeley, CA, USA, 2008) and PostGIS 2.4 (The PostGIS Development Group, Refractions Resarch Inc. Victoria, BC, Cananda, 2008). Spatial data were created in R (RStudio v 1.1.442, Boston, MA, USA). 

#### 2.3.2. Merging Accelerometry, GPS and Location Data

GPS data with the derived location of activity (within or outside the neighborhood) and accelerometry data were aggregated to the minute-level and matched based on the closest date and time stamp using SAS statistical software. If more than 50% of the GPS points within the minute were within a location (e.g., neighborhood) then the minute was categorized as within that location. Ultimately, a single dataset was generated for each study participant that contained minute-level accelerometry data and location (binary variables denoting within or outside the neighborhood). Only raw device data were used, and no GPS or accelerometry data were imputed. 

#### 2.3.3. Identification of Accelerometer Wear and Accelerometer-GPS Co-Wear

Sleep time and accelerometer non-wear were identified using a previously-published algorithm [24,25]. Logs were not used to track reasons for non-wear (e.g., water-based activities); therefore, all non-wear was treated the same. In brief, after the total sleep time was identified, periods of accelerometer non-wear were identified as sequences of at least 20 consecutive minutes of 0 activity counts. Waking wear time encompassed all minutes in the minute-level accelerometry data not identified as being part of the total sleep time or non-wear. Waking minutes with accelerometer wear and matched GPS data were classified as ‘co-wear’ minutes. 

#### 2.3.4. Measurement of Physical Activity

Average accelerometer counts per minute (CPM) were used as a measure of physical activity. Evenson cut-points were used to define moderate (≥2296–4011 CPM), vigorous (≥4012 CPM) and MVPA (≥2296 CPM) [26]. These cut-points show the best performance across intensity levels and are appropriate for adolescents [27,28]. The number of minutes per day in different intensities was determined by summing all minutes where the CPM was within the threshold and where the minutes were considered as accelerometer wear or as accelerometer-GPS co-wear (see description of analysis in Table 1).

#### 2.3.5. Sample Restrictions and Co-Wear Criteria

Of the 342 adolescents participating in TIGER Kids, 299 (87.4%) contributed accelerometry data (after 36 adolescents were asked to re-wear the device). TIGER Kids enrolled and measured participants year-round, but the current analysis is limited to adolescents who contributed accelerometry and GPS measurements outside of the summer holiday (*n* = 199; 66.6% of those who contributed accelerometry data). As we focus on physical activity occurring outside of school, the different weekday wear time patterns across the school year versus summer precluded pooling summer and school year data for the purpose of the current study. Adolescents assessed during the school year were more likely to be female (60.3% vs. 43.0% during the summer; *p* = 0.0046); otherwise, no differences were noted between those dropped versus retained sample for analysis. An additional 12 participants that reported a race other than white or African-American were excluded because this group was too small to draw comparisons. The current study is limited to 187 adolescents (54.7% of the original sample) who met minimum accelerometry wear time criteria of at least 3 days (including 1 weekend day) of 10 h per day of wear time (not including sleep or non-wear time; Table 1), who were assessed during the school year, and who reported being white or African-American race [14].

Three co-wear criteria (Table 1) were developed and applied that modeled minimum, moderate and stringent criteria based on a narrative review of the literature published since 2015 examining location-based physical activity among youth [11,16,29,30,31,32,33,34,35,36] and a similar article [22]. Across all co-wear criteria, a valid day was required to have ≥10 h of accelerometry wear. In the minimum co-wear criteria, the person was included if they had ≥2 valid weekdays and ≥1 valid weekend days of accelerometry wear and ≥180 min (3+ h) of co-wear across valid accelerometer days. In the moderate co-wear criteria, the person was included if they had ≥2 valid weekdays and ≥1 valid weekend days of accelerometry wear and ≥2 weekdays with ≥2 h after-school co-wear and ≥5 h on a weekend with co-wear. For both minimum and moderate co-wear criteria, all valid days for a valid person, regardless of number of minutes of accelerometer wear/GPS co-wear on a given day, or number of days, were analyzed. The stringent co-wear criteria is the only criteria that included GPS requirements for a valid day. In the stringent criteria, a valid day had to include ≥10 h of accelerometry wear and ≥3 h of after-school co-wear for a weekday or ≥7 h of co-wear for a weekend day. A person was included if they had ≥2 valid weekdays and ≥1 valid weekend day. All valid days for a valid person were analyzed.

### 2.4. Data Analysis

Analyses considered accelerometer wear and accelerometer/GPS co-wear that occurred during out-of-school hours (weekends and between the hours of 3:00 p.m. and 9:00 p.m. on weekdays) within the school year. Across wear time criteria, only activity that occurred during co-wear was analyzed. To evaluate differences across the three co-wear criteria as well as the accelerometry-only group, the four samples defined by wear time criteria were combined into a single dataset with a categorical predictor indicating membership in the criteria sample. Demographic differences across the four wear time criteria (Table 2) were assessed using linear models or chi-squared tests (person as the unit of analysis). Differences in average daily minutes of MVPA (occurring during co-wear) across the criteria (Table 3) were similarly assessed using linear models (person-day as unit of analysis). Differences in associations between MVPA occurring during co-wear with race, sex, weight status, and physical activity location across the criteria (Table 3) were assessed using linear models applied to a dataset that combined all three samples and which included a categorical predictor indicating membership in the co-wear criteria sample. Repeated-measures analysis (PROC GENMOD; SAS analytic software v 9.4, Cary, NC, USA) accounted for the presence of individuals across multiple criteria and adjusted for race, sex, weight category, age, weekday vs weekend, and wear time (minutes), as well as one-way interactions with the criteria variable to test for differences across the wear time criteria. Supplemental analyses considered moderate physical activity and vigorous physical activity as separate outcomes.

## 3. Results

On average participants had 17.3 daily MVPA minutes outside of school hours. Compared to the adolescents meeting the minimum accelerometer criteria (*n* = 187, 100%), nearly all adolescents (*n* = 174, 93.0%) also met the minimum co-wear criteria, whereas 142 participants (75.9%) and 128 participants (68.4%) met the moderate and stringent co-wear criteria, respectively (Table 2). Demographics (i.e., age, sex, race, weight category) did not vary significantly across the three samples (Table 2). 

Overall and within all of the subgroups considered (white adolescents, African-American adolescents, girls, boys, normal weight adolescents, and adolescents with overweight/obesity), adjusted mean minutes of MVPA did not differ significantly across the samples (Table 3). Similarly, estimates of location-based physical activity were similar across the samples. Despite the lack of significant subgroup-specific mean differences in physical activity across the four samples, there were significant differences across the samples in associations. In the minimum and moderate criteria groups there was no association between race and physical activity, whereas in the most stringent group there was a significant association, with African Americans having significantly higher MVPA compared to whites (*p* = 0.0233); the association between race and MVPA differed across the four criteria-based samples (*p* = 0.0110). Across all four criteria, boys had higher levels of MVPA and participants with overweight/obesity had lower levels of MVPA; however, the magnitude of these associations differed across the criteria (sex and MVPA, *p* = 0.0409; weight status and MVPA, *p* = 0.0103). Patterns were similar for moderate physical activity and vigorous physical activity, when modeled separately (Supplemental Tables S1 and S2). 

## 4. Discussion

The physical activity field lacks justification for and consistent use of accelerometry and GPS co-wear criteria. This study aimed to describe the impact of applying different co-wear criteria among a diverse group of adolescents in Baton Rouge, Louisiana. Estimates of overall and location-based physical activity were consistent across the criteria-based samples even within sociodemographic subgroups. However, associations between physical activity and sex, race, and weight status differed across the co-wear criteria samples.

Guidance exists for analysis of accelerometry data, including what represents a ‘valid day’ of wear time and what represents a ‘valid person’ for analysis [28], which ensures consistency across studies. However, similar guidance does not currently exist that incorporates wear time of GPS units to capture the location of physical activity. To date, only one study has examined the impact of different GPS criteria on sample characteristics [22], finding sociodemographic differences among the retained sampled compared to those excluded from analysis. In the current study, we did not identify systematic differences in participant age, race, gender, or weight status across the different co-wear criteria-based samples. This could be due to the fact that in our sample even the most stringent criteria retained 68% of the sample, which was higher than the percentage retained in Mavoa et la., 2018 (34% and 19% of full sample for moderate and stringent criteria, respectively) [22]. This may be credited to researchers using strategies to minimize data loss, including providing participants with clear instructions and ongoing support, sending participants regular reminder messages to charge their devices, providing letters to inform schools of devices and providing incentives to participants. It is critical that researchers use these strategies to ensure that inclusion criteria do not generate large sample size reductions that may result in sociodemographic differences, and potential environmental biases, as demonstrated by Mavoa et al., 2018 [22].

We found that the different co-wear criteria, however, produced differing conclusions about associations with physical activity. If we use the accelerometry-only group as a gold standard for associations, the sample generated from the strictest co-wear criterion was more likely to produce associations substantially different from the gold standard accelerometry-only sample. For example, the stringent criteria sample is the only one to find a significant association between race and physical activity, with African-Americans having significantly higher physical activity compared to whites. Furthermore, the largest differences in physical activity according to weight status were found in the stringent criteria sample. These differences may be due to unmeasured factors related to compliance (e.g., self-selection of motivated individuals), but also demonstrates the critical impact co-wear criteria may have on conclusions drawn from research examining health disparities. This provides new insight into GPS wear time criteria, extending and building upon earlier research that advocated for research developing and standardizing accelerometry wear time criteria [19,20,21].

Our findings have limitations, yet highlight potential issues for future studies. Findings are sample-specific and limited by low overall (17 min/day of MVPA) and location-specific (8 min/day within neighborhood and 10–11 min/day outside of neighborhood) minutes of MVPA and small standard error that limited our ability to detect differences in location-based physical activity, especially within subgroups. Furthermore, this study did not explore other environmental attributes or location-based physical activity (e.g., park-based activity) that may demonstrate important biases across samples.

## 5. Conclusions

Standardizing the analysis of accelerometer-GPS co-wear data is critical to minimize measurement bias and provide a uniform platform to compare results within and between populations and studies [37]. This study demonstrates that inconsistent co-wear criteria may impact conclusions drawn from research. Additional research and understanding of ideal co-wear criteria are needed to reduce bias and accurately estimate free-living location-based physical activity. While criteria may vary depending on the research question, it is important to disseminate evidence to support ideal criteria to standardize the analysis of accelerometer–GPS co-wear data when appropriate.

## Figures and Tables

**Table 1:** Descriptions of wear time criteria and number of participants and participant/days meeting the various criteria.

			Accelerometer Only ^1^	Accelerometer + Minimum GPS Co-Wear	Accelerometer + Moderate GPS Co-Wear	Accelerometer + Stringent GPS Co-Wear
**Valid day**	Accelerometry		≥10 h accelerometer wear	≥10 h accelerometer wear	≥10 h accelerometer wear	≥10 h accelerometer wear
	GPS co-wear	Weekday	--	--	--	≥3 h of after-school co-wear
		Weekend	--			≥7 h of co-wear
**Valid person** ^2^	Accelerometry	Weekday	≥2 valid weekdays	≥2 valid weekdays	≥2 valid weekdays	≥2 valid weekdays and ≥1 valid weekend day
		Weekend	≥1 valid weekend days	≥1 valid weekend days	≥1 valid weekend days	
	GPS co-wear	Weekday	--	≥180 min (3+ h) of co-wear across valid accelerometer days	≥2 weekdays with ≥2 h after-school co-wear	
		Weekend	--		≥5 h of co-wear	
**Analysis**			Analyzed all days with valid accelerometer wear. Analyzed accelerometry for all minutes of accelerometer wear, regardless of co-wear.	Analyzed all valid days for a valid person, regardless of number of minutes of accelerometer wear/GPS co-wear on a given day, or number of days. As accelerometry was analyzed only for co-wear minutes, days with 0 min of co-wear were excluded from analysis.	Analyzed all valid days for a valid person, regardless of number of minutes of accelerometer wear/GPS co-wear on a given day, or number of days. As accelerometry was analyzed only for co-wear minutes, days with 0 min of co-wear were excluded from analysis.	Analyzed all valid days for a valid person. Analyzed accelerometry-only for co-wear minutes.
**Persons (*n*)**			187	174	142	128
**Person-days (*n*)**			1346	953	840	703

^1^ Participants were required to meet the minimum accelerometry wear time criteria. Therefore, this reflects the entire sample. ^2^ Determination of a ‘valid person’ only considers ‘valid days’ as input.

**Table 2:** Demographic and co-wear characteristics across samples resulting from different GPS-accelerometer co-wear criteria.

	Accelerometer Only ^1^	Accelerometer + GPS Wear Time Criteria			
		Accelerometer + Minimum Co-Wear	Accelerometer + Moderate Co-Wear	Accelerometer + Stringent Co-Wear	*p*-Value ^2^
Persons (*n*)	187	174	142	128	
Age, mean (SD)	12.3 (1.9)	12.3 (1.9)	12.2 (1.9)	12.1 (1.9)	0.7427
Sex, *n* (%)					
Female	111 (59.4)	102 (58.6)	79 (55.6)	74 (57.8)	0.8624
Male	76 (40.6)	72 (41.4)	63 (44.4)	54 (42.2)	
Race, *n* (%)					0.7687
White	115 (61.5)	110 (63.2)	93 (65.5)	86 (67.2)	
AA	72 (38.5)	64 (36.8)	49 (34.5)	42 (32.8)	
Weight category, *n* (%)					0.9995
≤Normal Weight	84 (44.9)	82 (47.1)	64 (45.1)	60 (46.9)	
Overweight	28 (15.0)	27 (15.5)	23 (16.2)	21 (16.4)	
Obese	42 (22.5)	39 (22.4)	33 (23.2)	27 (21.1)	
Severely obese	33 (17.7)	26 (14.9)	22 (15.5)	20 (15.6)	
GPS co-wear (out-of-school) ^3,4^, *n* (%)					
0%	13 (7.0)	--	--	--	
0.1–39.9%	26 (13.9)	26 (14.9)	8 (5.6)	5 (3.9)	
40–69.9%	70 (37.4)	70 (40.2)	58 (40.9)	47 (36.7)	
70–89.9%	30 (16.0)	30 (17.2)	28 (19.7)	28 (21.9)	
≥90%	48 (25.7)	48 (27.6)	48 (33.8)	48 (37.5)	

^1^ Study participants having at least 2 weekdays and 1 weekend day of 10+ h of non-sleep accelerometer wear time; ^2^ Differences across samples assessed via linear models or chi-squared tests (person as unit of analysis); ^3^ Co-wear (by person): % of all out-of-school accelerometry minutes with GPS data, including across days with 0 min of co-wear; ^4^ Out-of-school time was defined as being on a weekend, or between 3:00 p.m. and 9:00 p.m. on weekdays.

**Table 3:** Differences in estimated daily minutes of moderate-to-vigorous physical activity and associations with race, sex, and physical activity location across datasets resulting from different GPS–accelerometer co-wear criteria.

	Accelerometer Only	Accelerometer + GPS Wear Time Criteria			*p*-Value ^a^
		Minimum Co-Wear	Moderate Co-Wear	Stringent Co-Wear	
Persons (*n*)	187	174	142	128	
Person-days (*n*)	1346	953	840	703	
Daily MVPA mins (out-of-school), mean (SE)	17.3 (1.5) ^b^	18.6 (1.0) ^b^	18.8 (1.1) ^b^	18.2 (1.5) ^b^	0.7248
Race					
White (ref)	19.2 (1.6) ^c^	18.8 (1.2) ^c^	18.6 (1.2) ^c^	17.2 (1.4) ^c^	0.3222
AA	20.4 (2.1)	20.5 (1.5)	21.5 (1.8)	23.0 (2.6)	0.3156
*b* (*se*)	*1.3* (*1.9*)	*1.7* (*1.8*)	*2.9* (*2.1*)	*5.7* (*2.5*) *	*0.0110*
Sex					
Girls (ref)	16.7 (1.7) ^c^	17.3 (1.1) ^c^	17.3 (1.2) ^c^	17.0 (1.8) ^c^	0.9389
Boys	22.9 (2.0)	22.0 (1.5)	22.8 (2.1)	23.2 (2.1)	0.3009
*b* (*se*)	*6.1* (*1.8*) ***	*4.7* (*1.7*) **	*5.5* (*1.8*) **	*6.2* (*2.1*) **	*0.0409*
Weight status					
≤Normal Weight (ref)	23.9 (2.2) ^c^	23.6 (1.6) ^c^	24.4 (1.9) ^c^	25.8 (2.4) ^c^	0.3762
Overweight/obese	15.7 (1.6)	15.7 (1.1)	15.8 (1.2)	14.4 (1.7)	0.5503
*b* (*se*)	*−8.1* (*2.1*) ***	*−7.9* (*1.9*) ***	*−8.6* (*2.2*) ***	*−11.4* (*2.5*) ***	*0.0132*
Location ^d^					
Inside neighborhood buffer (ref)		8.3 (0.6) ^c^	8.5 (0.7) ^c^	8.5 (0.8) ^c^	0.5953
Outside neighborhood buffer		10.8 (0.7)	11.0 (0.8)	11.3 (0.9)	0.2924
*b* (*se*)		*2.5* (*0.9*) **	*2.5* (*1.0*) **	*2.7* (*1.1*) *	*0.6821*

^a^*p*-values resulting from tests for differences across samples; ^b^ Least-squares estimates from models that adjust for weekday/weekend and wear (accelerometer only) or co-wear (co-wear criteria) time; ^c^ Least square estimates result from models that adjust for race, sex, weight status, age, weekday/weekend, and (daily) wear time; ^d^ For the models investigating location of PA, wear time was the location-specific wear time vs total (daily) wear time; *b* (*se*) * *p* < 0.05, ** *p* < 0.01, *** *p* < 0.001.

## Data Availability

The datasets used and analyzed during the current study are available from the corresponding author on reasonable request.

## Supplementary Materials

The following supporting information can be downloaded at: https://www.mdpi.com/article/10.3390/ijerph19105931/s1, Table S1: Differences in estimated minutes of moderate physical activity and associations with race, sex, and physical activity location across datasets resulting from different GPS-accelerometer co-wear criteria; Table S2: Differences in estimated minutes of vigorous physical activity and associations with race, sex, and physical activity location across datasets resulting from different GPS-accelerometer co-wear criteria.
