# Supplementary material for: The Potential for Bias across GPS-Accelerometer Combined Wear Criteria among Adolescents
Source: Int J Environ Res Public Health. 2022 May 13;19(10):5931. doi: 10.3390/ijerph19105931 (PMC9141158; doi:10.3390/ijerph19105931)
Supplement: Supplementary file 1 [file ijerph-19-05931-s001.zip › ijerph-1629812-supplementary.pdf]

Supplementary Table S1. Differences in estimated minutes of moderate physical activity and associations with race, sex, and physical activity location across datasets resulting from different GPS-accelerometer co-wear criteria

	Accelerometer Only	Accelerometer+GPS Wear-Time Criteria			*p*-value [a]
		Minimum Co-Wear	Moderate Co-Wear	Stringent Co-Wear	
Persons (n)	187	174	142	128	
Person-days (n)	1,346	953	840	703	
Daily MPA mins (out of school), mean (SE)	12.3 (1.0) [b]	13.2 (0.6) [b]	13.2 (0.7) [b]	12.6 (0.9) [b]	0.4622
Race					
White (ref)	13.3 (1.0) [c]	13.4 (0.7) [c]	13.2 (0.7) [c]	12.2 (0.9) [c]	0.2911
AA	14.1 (1.2)	14.0 (0.9)	14.6 (1.0)	15.1 (1.4)	0.3881
b (se)	*0.8 (1.2)*	*0.6 (1.0)*	*1.3 (1.2)*	*3.0 (1.4)**	*0.0094*
Sex					
Girls (ref)	12.0 (1.1) [c]	12.3 (0.7) [c]	12.3 (0.7) [c]	12.0 (1.0) [c]	0.9586
Boys	15.4 (1.2)	15.0 (0.9)	15.5 (0.9)	15.3 (1.2)	0.2763
b (se)	*3.4 (1.1)***	*2.7 (1.0)***	*3.1 (1.1)***	*3.3 (1.2)***	*0.1041*
Weight status					
≤Normal Weight (ref)	15.4 (1.3) [c]	15.1 (0.9) [c]	15.5 (1.0) [c]	16.1 (1.3) [c]	0.5716
Overweight/obese	12.0 (1.1)	12.2 (0.7)	12.2 (0.8)	11.2 (1.1)	0.4631
b (se)	*-3.4 (1.2)***	*-3.0 (1.1)***	*-3.3 (1.3)**	*-4.9 (1.4)***	*0.0202*
Location [d]					
Inside neighborhood buffer (ref)		5.9 (0.3) [c]	5.9 (0.4) [c]	5.9 (0.5) [c]	0.7255
Outside neighborhood buffer		7.3 (0.4)	7.5 (0.4)	7.5 (0.5)	0.4149
b (se)		*1.5 (0.5)***	*1.5 (0.6)***	*1.6 (0.7)**	*0.8109*

[a] P-values resulting from tests for differences across samples.
[b] Least squares estimates from models that adjust for weekday/weekend and wear (accelerometer only) or co-wear (co-wear criteria) time.
[c] Least square estimates result from models that adjust for race, sex, weight status, age, weekday/weekend, and (daily) wear time.
[d] For the models investigating location of PA, wear time was the location-specific wear time vs total (daily) wear time.
b (se) * $p<0.05$ ** $p<0.01$

Supplementary Table S2. Differences in estimated minutes of vigorous physical activity and associations with race, sex, and physical activity location across datasets resulting from different GPS-accelerometer co-wear criteria

	Accelerometer Only	Accelerometer+GPS Wear-Time Criteria			*p*-value [a]
		Minimum Co-Wear	Moderate Co-Wear	Stringent Co-Wear	
Persons (n)	187	174	142	128	
Person-days (n)	1,346	953	840	703	
Daily VPA mins (out of school), mean (SE)	5.0 (0.7) [b]	5.5 (0.5) [b]	5.6 (0.5) [b]	5.5 (0.8) [b]	0.8771
Race					
White (ref)	5.8 (0.7) [c]	5.4 (0.5) [c]	5.4 (0.6) [c]	5.0 (0.6) [c]	0.5985
AA	6.3 (1.1)	6.6 (0.9)	7.0 (1.1)	7.9 (1.6)	0.4330
b (se)	*0.5 (1.0)*	*1.1 (1.1)*	*1.6 (1.3)*	*2.8 (1.6)*	*0.0787*
Sex					
Girls (ref)	4.7 (0.8) [c]	5.0 (0.6) [c]	5.0 (0.7) [c]	5.0 (1.0) [c]	0.9206
Boys	7.4 (1.0)	7.0 (0.7)	7.4 (0.8)	7.9 (1.1)	0.3497
b (se)	*2.7 (0.9)***	*2.0 (0.9)**	*2.3 (0.9)**	*2.9 (1.1)***	*0.0196*
Weight status					
≤Normal Weight (ref)	8.4 (1.1) [c]	8.5 (1.0) [c]	8.9 (1.2) [c]	9.7 (1.5) [c]	0.3904
Overweight/obese	3.7 (0.7)	3.5 (0.4)	3.5 (0.5)	3.2 (0.7)	0.8293
b (se)	*-4.7 (1.1)****	*-4.9 (1.1)****	*-5.3 (1.3)****	*-6.6 (1.5)****	*0.0321*
Location [d]					
Inside neighborhood buffer (ref)		2.4 (0.3) [c]	2.5 (0.3) [c]	2.6 (0.4) [c]	0.3525
Outside neighborhood buffer		3.4 (0.4)	3.5 (0.5)	3.8 (0.6)	0.1887
b (se)		*1.0 (0.5)**	*1.0 (0.5)**	*1.1 (0.6)*	*0.3201*

[a] P-values resulting from tests for differences across samples.
[b] Least squares estimates from models that adjust for weekday/weekend and wear (accelerometer only) or co-wear (co-wear criteria) time.
[c] Least square estimates result from models that adjust for race, sex, weight status, age, weekday/weekend, and (daily) wear time.
[d] For the models investigating location of PA, wear time was the location-specific wear time vs total (daily) wear time.
b (se) * p<0.05 ** p<0.01 *** p<0.001
